# Delivery and assessment of a CRISPR/nCas9-based genome editing system on in vitro models of mucopolysaccharidoses IVA assisted by magnetite-based nanoparticles

**DOI:** 10.1038/s41598-022-19407-x

**Published:** 2022-09-03

**Authors:** Andrés Felipe Leal, Javier Cifuentes, Carlos Emilio Torres, Diego Suárez, Valentina Quezada, Saúl Camilo Gómez, Juan C. Cruz, Luis H. Reyes, Angela Johana Espejo-Mojica, Carlos Javier Alméciga-Díaz

**Affiliations:** 1 Institute for the Study of Inborn Errors of Metabolism, Faculty of Science, Pontificia Universidad Javeriana, Cra. 7 No. 43-82 Building 54, Room 305A, 110231 Bogotá D.C., Colombia; 2 Department of Biomedical Engineering, Universidad de los Andes, 111711 Bogotá, Colombia; 3 Grupo de Diseño de Productos y Procesos (GDPP), Department of Chemical and Food Engineering, Universidad de los Andes, 111711 Bogotá, Colombia

**Keywords:** Gene therapy, Nanobiotechnology

## Abstract

Mucopolysaccharidosis IV A (MPS IVA) is a lysosomal disorder caused by mutations in the *GALNS* gene. Consequently, the glycosaminoglycans (GAGs) keratan sulfate and chondroitin 6-sulfate accumulate in the lysosomal lumen. Although enzyme replacement therapy has shown essential advantages for the patients, several challenges remain to overcome, such as the limited impact on the bone lesion and recovery of oxidative profile. Recently, we validated a CRISPR/nCas9-based gene therapy with promising results in an in vitro MPS IVA model. In this study, we have expanded the use of this CRISPR/nCas9 system to several MPS IVA fibroblasts carrying different *GALNS* mutations. Considering the latent need to develop more safety vectors for gene therapy, we co-delivered the CRISPR/nCas9 system with a novel non-viral vector based on magnetoliposomes (MLPs). We found that the CRISPR/nCas9 treatment led to an increase in enzyme activity between 5 and 88% of wild-type levels, as well as a reduction in GAGs accumulation, lysosomal mass, and mitochondrial-dependent oxidative stress, in a mutation-dependent manner. Noteworthy, MLPs allowed to obtain similar results to those observed with the conventional transfection agent lipofectamine. Overall, these results confirmed the potential of CRISPR/nCas9 as a genome editing tool for treating MPS IVA. We also demonstrated the potential use of MLPs as a novel delivery system for CRISPR/nCas9-based therapies.

## Introduction

Mucopolysaccharidosis IVA (MPS IVA, Morquio syndrome A, OMIM #253000) is a rare genetic disease caused by impaired *N*-acetylgalactosamine-6-sulfatase (GALNS, E.C.3.1.6.4) activity, which led to the lysosomal accumulation of keratan sulfate (KS) and chondroitin 6-sulfate (C6S)^1,2^. Under impaired GALNS activity, among many others, several pathological signal pathways related to oxidative stress^3,4^ and inflammation^5–7^ might take place. Although non-skeletal findings are found in MPS IVA patients, skeletal dysplasia constitutes the most common alteration^8^. MPS IVA has a global prevalence ranging from 0.07 to 3.62 per 100,000 population^9^, while in Colombia is the most prevalent MPS (1.98 per 100,000 population)^9,10^.

Currently, the enzyme replacement therapy (ERT), which consists of the weekly administration of elosulfase alfa (Vimizim), given its low half-life, fails to reach therapeutic effect on the bone manifestations of the disease. Since mutations in the *GALNS* gene are the primary cause of MPS IVA, gene therapy (GT) continues to be a feature as the most promising strategy^1,11^. Although some classical GT-based approaches have been attempted on preclinical assays^12,13^, they have not implemented in the clinical practice yet. In addition, several challenges associated with the transient transgene expression and the inherent risk of the use of viral vectors need to be solve to reach a successful therapy^14,15^.

Some novel strategies based on genome editing could overcome the current challenges of the classical GT. For instance, the clustered, regularly interspaced short palindromic repeats and CRISPR-associated protein 9 (CRISPR/Cas9) have emerged as a valuable nuclease-based genome editing mechanism given its straightforward design, cost-effectiveness, and high specificity^16^. In addition, Cas9 variants, such as Cas9 nickase (nCas9), have shown an increased efficiency in the *On-target* cut, as well as a decreased *Off-target* effect^17^. This nCas9, that contains a mutation in the RuvC domain (D10A)^17^, requires two sgRNA to target Cas9 to a specific genomic region and mediate the double-strand break. For genome editing purposes, if a concomitant donor template containing homologous recombination arms is delivered, a high occurrence of homologous recombination will take place to introduce foreign genetic information into the nuclear genome through *knock-in* approaches^16,18^.

In this scenario, we recently reported on the suitability of CRISPR/nCas9-based gene therapy as a potential approach to treat MPS IVA in vitro with undetectable *Off-target* effects and the recovery of the significant pathological biomarkers to wild-type levels^11^. CRISPR/Cas9-based systems have also been used for other lysosomal storage disorders (LSD), with encouraging results^19–27^. Despite the well-known limitations of the viral vectors, they are widely used because of their natural capacity for transduction^8,28,29^. However, novel non-viral engineered vectors could overcome some of the classical challenges of the viral ones, which include limited size packing, random DNA integration, and immune response activation, among others^30^. In previous work, we developed a novel cell-penetrating vector based on core–shell, magnetite-silver nanoparticles pH-responsive magnetite nanoparticles (MNP)-based carrier for nucleic acids (MNPs@Ag), capable of forming complexes with nucleic acids through a pH-responsive polymer (pDMAEMA) (MNPs@Ag-pD). Escape from the endolysosomal degradation pathway by the presence of the membrane translocating peptide Buforin II (MNPs@Ag-pD/BUF-II) was also observed^31,32^. These nanobioconjugates were entrapped in liposomes forming magnetoliposomes (MLPs) to prevent the potential DNA degradation by extracellular nucleases^31,33^.

To test the usefulness of the MLPs as potential carriers of the CRISPR/nCas9 system, in this study we conducted in vitro experiments on MPS IVA fibroblasts containing different mutations of the *GALNS* gene. We evaluated the effect of the MLPs-coupled CRISPR/nCas9 system by using key biomarkers such as GAGs accumulation, lysosomal mass, and mitochondrial-dependent oxidative stress (mtROS).

## Results

### Magnetoliposome endocytosis-mediated uptake is not cytotoxic and increases the transfection ratio on primary MPS IVA fibroblasts

**Figure 1:**
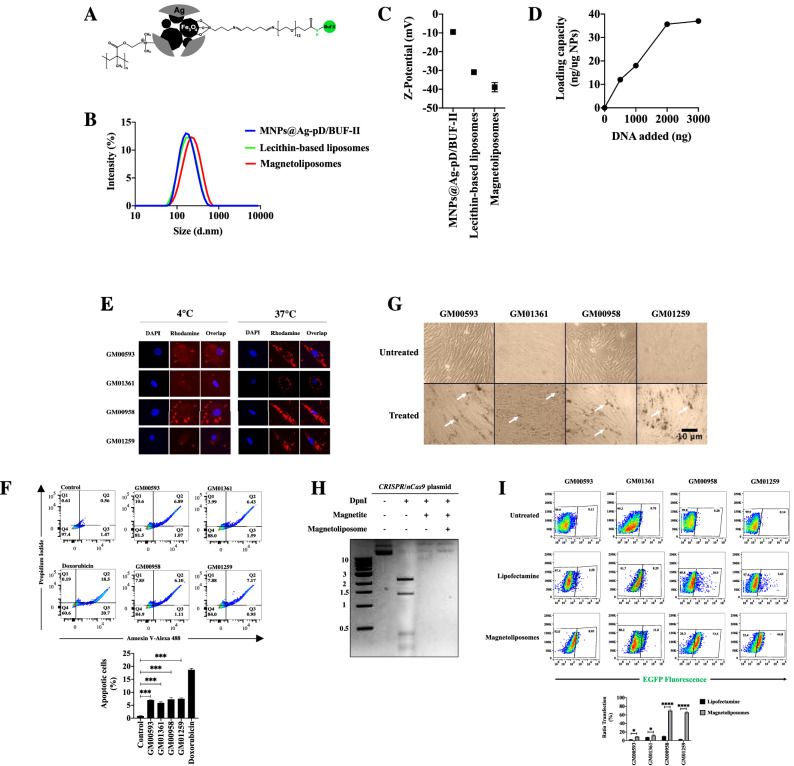
MLPs internalization and their impact on the MPS IVA fibroblast viability. (**A**) Schematic representation of the MNPs@Ag-pD/BUF-II nanobioconjugate. (**B**) Size distribution determined by DLS. Note the increased size of MLPs (red line) respect, MNPs@Ag-pD/BUF-II (line blue), and lecithin-based liposomes (yellow line). (**C**) Zeta-potential of the MNPs@Ag-pD/BUF-II, lecithin-based liposomes, and MLPs. (**D**) MNPs@Ag-pD/BUF-II DNA loading capacity. Note a linear DNA loading capacity up to 2000 ng. (**E**) Internalization assays. Cells were incubated with Rho-MLPs for 4 h at 4 °C and 37 °C. Blue and red fluorescence correspond to the nucleus and MNPs@Ag-pD/BUF-II. For 4 °C incubation, the cells were overexposed in the red channel to observe easier the cytoplasmic region. Notice the peripheral accumulation of MNPs@Ag-pD/BUF-II at 4 °C, while at 37 °C, most MNPs were present inside the MPS IVA fibroblasts. (**F**) Apoptosis assay on MPS IVA fibroblasts incubated with MLPs. The upper panel shows a representative double quadrant flow cytometer draw. The mean of three independent experiments is shown in the bottom panel. Doxorubicin was used as a positive control of apoptosis. (**G**) DIC of MPS IVA fibroblasts incubated for 4 h with MLPs. No evident loss of confluence or morphological changes were observed. White arrows show MNPs@Ag-pD/BUF-II accumulation. (**H**) Agarose gel for *DpnI* protection assay. Note that using either MNPs@Ag-pD/BUF-II or MLPs, the plasmid donor was not degraded after *Dpn*I incubation. An unprocessed image is shown in Supplementary Fig. 2. (**I**) Transfection efficiency of LP- and MLPs-conjugated Donor GALNS:AAVS1 on MPS IVA fibroblasts. A representative cytogram for GFP fluorescence is presented on the upper panel, while the bottom panel shows the mean for four independent assays. *p ≤ 0.05, ***p ≤ 0.001, ****p ≤ *0.0001*. Two-tailed Student's t-test.

Magnetoliposomes used in this study are made up of a magnetic nanoparticle (MNPs@Ag-pD/BUF-II, Fig. 1A) and a lecithin-based liposome (see “Materials and methods”). The initial characterization showed an average hydrodynamic diameter of 195 nm for MNPs@Ag-pD/BUF-II, and 197 nm for lecithin-based liposomes (Fig. 1B). In comparison, MLP increased up to 247 nm (Fig. 1B). For Z-potential, we observed MNPs@Ag-pD/BUF-II as the less negative (− 9.5 ± 0.9 mV), followed by lecithin-based liposomes (− 30.9 ± 0.5 mV), and the MLPs (− 38.9 ± 2.4 mV) (Fig. 1C). Finally, the loading DNA capacity was calculated as 36 ng DNA/μg MNPs@Ag-pD/BUF-II (Fig. 1D).

Regarding internalization assays, the incubation of 25 μg Rhodamine B-labeled MLPs (Rho-MLPs) at 37 °C but not at 4 °C resulted in an observable intracellular red fluorescence signal in all the four MPS IVA fibroblasts (Fig. 1E). Previously, we demonstrated that our MLPs exhibited high cytocompatibility (above 80%) in Vero cells^31^. Given that primary cells are more sensitive than cell lines^34^, we determined the cytotoxic/proapoptotic effect of MLPs on MPS IVA fibroblasts after 48 h of interaction. Only ~ 8% of late-apoptotic cells were observed after treatment with MLPs (Fig. 1F). Similarly, more than 80% of viability was determined by MTT and LDH assays (Supplementary Fig. 3). Likewise, we failed to detect evident morphological changes under DIC microscopy (Fig. 1G). Liposomes and functionalized core–shell nanoparticles showed similar low cytotoxicity and negligible impact on morphology when evaluated as single formulations (data not shown).

Previously, it was demonstrated that MNPs@Ag-pD/BUF-II could escape from the endosomal pathway (due to the conjugated of Buforin II peptide) and that they can charge/discharge plasmid vectors^31^. To evaluate the potential protecting effect of vehicles against endonuclease-dependent degradation plasmids, we carried out a restriction enzyme assay using *Dpn*I in the presence of MNPs@Ag-pD/BUF-II and MLPs-attached CRISPR/nCas9 plasmids. Although a slight band was observed for either MNPs@Ag-pD/BUF-II or MLPs, those were at the same molecular weight as untreated DNA, suggesting that *DpnI* failed to digest the conjugated pDNA to MNPs@Ag-pD/BUF-II or making part of MLPs (Fig. 1H). Potential unknown interference between phenol:chloroform:isoamyl alcohol and MNP/MLPs could prevent the complete release of the coupled plasmids. Transfections assays on MPS IVA fibroblast using LP and MLPs were carried out using the Donor AAVS1:GALNS plasmid, which contains a GFP gene, to determine whether they can effectively deliver the CRISPR/nCas9-associated plasmids into mammalian cells. After 48 h post-interaction, an increase in the GFP signal was detected in all fibroblasts (Fig. 1I) using both LP and MLPs. Interestingly, compared with LP, the MLPs-transfection led to a statistically significant increase in the transfection ratio of 1.5 ± 0.2, 3.5 ± 0.5, 6.8 ± 0.3, and 21.1 ± 2.1-fold for the GM01361, GM00593, GM00958, and GM01259 fibroblasts, respectively. Overall, these results showed that MLPs are well-suited as carriers of CRISPR/Cas9-associated nucleic acids for effective intracellular delivery.

### Long-term CRISPR/nCas9-based genome editing is observed on LP and MLPs-transfected MPS IVA fibroblasts

**Figure 2:**
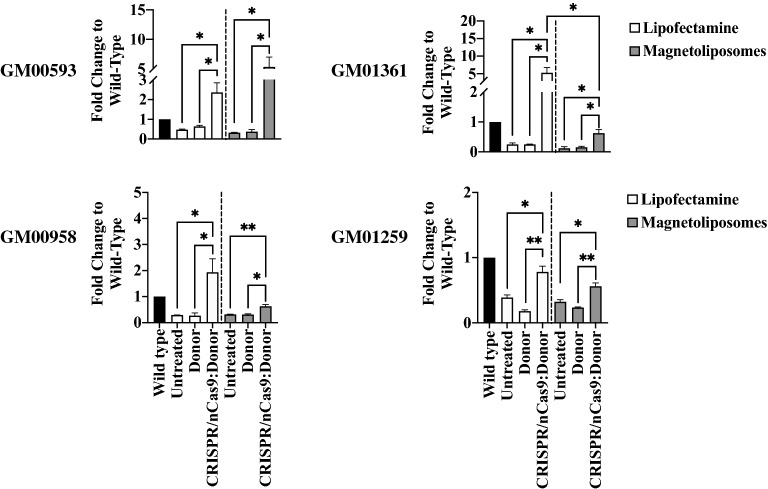
*GALNS* gene expression on long-term CRISPR/nCas9 treated fibroblasts. The relative transcription fold-change levels to wild-type for MPS IVA fibroblasts after transfection with LP (n = 4) and MLPs (n = 4)-conjugated CRISPR/nCas9 are shown. Fibroblasts were transfected either with the Donor AAVS1:GALNS plasmid (Donor) or the CRISPR/nCas9-Donor AAVS1:GALNS plasmids (CRISPR/nCas9:Donor). Note that LP allowed a higher transcription for GM01361 fibroblasts treated with CRISPR/nCas9 plus Donor AAVS1:GALNS levels compared to MLPs (*p* = *0.0382*). Fold-change corresponds to comparison between untreated cells in which a reduced mRNA levels were found respect to wild-type (established as 1) as follows: GM00593: 0.47 ± 0.035, GM01361: 0.25 ± 0.052, GM00958: 0.29 ± 0.002, GM01259: 0.3892 ± 0.038.

After LP- and MLPs-transfection, *GALNS* transcription and enzyme activity increased in all the fibroblast cells tested compared to untreated cells. Surprisingly, compared to wild-type, *GALNS* transcript levels increased in all the fibroblasts independently of the MLPs transfection ratio (Fig. 2). GM00593 fibroblasts had the highest mRNA levels for both LP (fold-change: 2.3 ± 0.5) and MLPs (fold-change: 5.3 ± 1.7) treatments (Fig. 2). We detected supraphysiological mRNA levels on GM01361 and GM00958 fibroblasts treated with LP. After MLPs treatment, these cells showed a significant mRNA increase compared to the untreated ones. In the case of GM01259 fibroblasts, untreated cells showed 40% of *GALNS* mRNA levels, compared with wild-type fibroblasts, with a slight increase after LP (fold-change: 0.78 ± 0.09) or MLPs (fold-change: 0.56 ± 0.05) treatments (Fig. 2).

**Figure 3:**
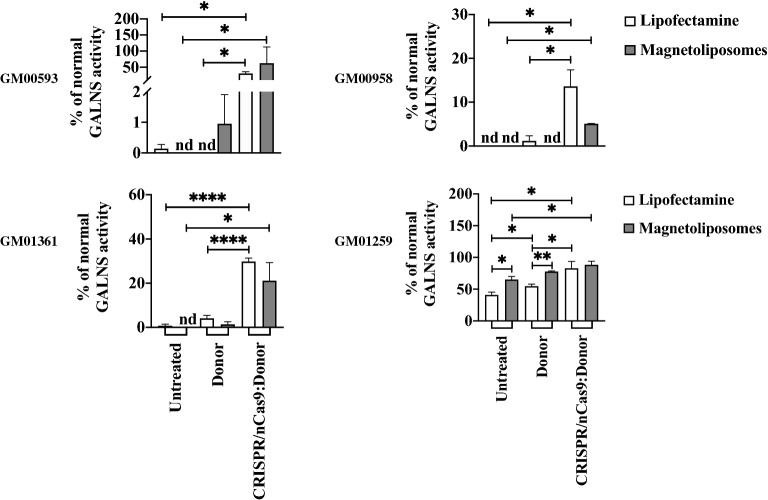
GALNS activity after long-term CRISPR/nCas9 treatment. The figure shows the GALNS activity to wild-type levels reached after one month of genome editing treatment with LP (n = 4) and MLPs (n = 3). Fibroblasts were transfected either with the Donor AAVS1:GALNS plasmid (Donor) or the CRISPR/nCas9-Donor AAVS1:GALNS plasmids (CRISPR/nCas9:Donor). Notice that GM00958 fibroblasts were the less responsive MPS IVA fibroblasts. *p ≤ 0.05, **p ≤ 0.005, ****p ≤ *0.0001*. GM00593 and GM00958 were analyzed using the Mann–Whitney U test and GM01361 and GM01259 with the Two-tailed Student's t-test.

Enzyme activity findings matched the profile observed for *GALNS* mRNA levels (Fig. 3). For instance, the GM00593 fibroblast showed the highest enzyme activity levels when transfected with LP (30.4 ± 5.25% WT levels, *p* = *0.0286*) and MLPs (62 ± 10.62% WT levels, *p* = *0.0286*). Likewise, GM01361 fibroblasts showed higher enzyme activity after transfection with LP (29.7 ± 1.63% WT levels, *p* ≤ *0.0001*) than with MLPs (21 ± 8.2% WT levels, *p* = *0.0276*) (Fig. 3), which agree well with the transcription assays.

Before transfection, GM01259 fibroblasts showed an enzyme activity of 41 ± 8.6% WT levels. Surprisingly, this enzyme activity increased to 65.1 ± 4.88% after incubating with uncharged MLPs (i.e. empty MLPs) (Fig. 3). After transfection, the GALNS activity in these fibroblasts significantly increased with both the LP (82.9 ± 10.74% WT levels, *p* = *0.0109*) and MLPs (88.4 ± 5.61% WT levels, *p* = *0.0348*). GM00958 fibroblasts exhibited the lowest increase in GALNS activity after treatment with LP (13 ± 3.78% WT levels, p = *0.0286*) and MLPs (5 ± 0.12% WT levels, *p* = *0.0286*); however, such levels were significantly higher compared to untreated cells (Fig. 3). We did not observe any significant increase in *GALNS* mRNA or GALNS activity in fibroblasts transfected only with the Donor AAVS1:GALNS plasmid, except for GM01259, in which GALNS activity increased ~ 14% with LP-coupled Donor AAVS1:GALNS, compared to untreated cells (*p* = *0.0452*).

Finally, an increase in the extracellular GALNS activity was observed for GM00593 with both transfection approaches (LP: 103.4 ± 25.61% WT levels, *p* = *0.0121*; MLPs: 64.3 ± 16.22% WT levels, *p* = *0.0487*) (Supplementary Fig. 4), whereas for GM01361, GM00958, and GM01259 no significant change in extracellular enzyme activity was observed (data not shown). These data provide further evidence of previous findings that suggest that CRISPR/nCas9 is a suitable genome editing tool for MPS IVA^11^. Also, they show the potential of MLPs as non-viral vectors for the transport and delivery of the CRISPR/nCas9 system, with results similar to those observed for a reference delivery system (i.e., lipofectamine).

### GAGs accumulation and lysosomal mass are positively impacted in a GALNS activity-dependent way

**Figure 4:**
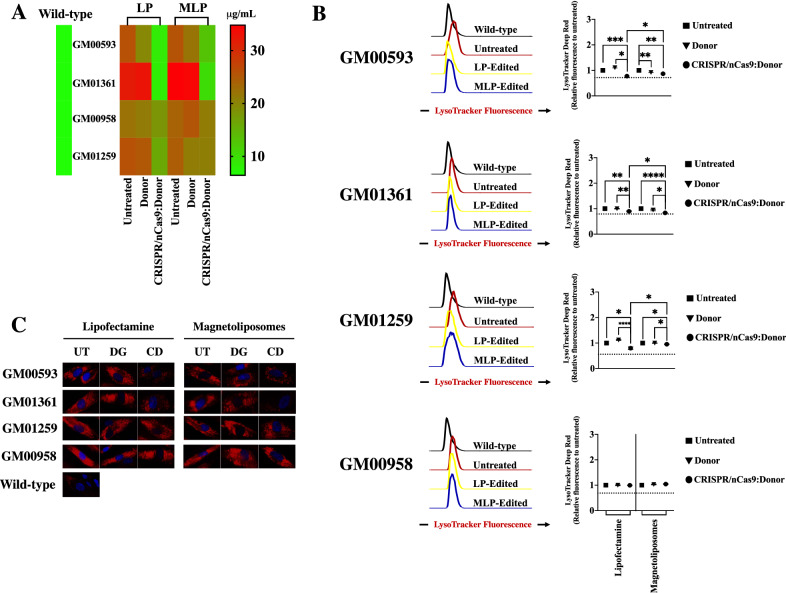
GAGs accumulation and lysosomal mass evaluation. (**A**) Heat map showing the global behavior of GAGs accumulation (n = 4). GM00593 and GM01361 fibroblasts showed the best recovery after CRISPR/nCas9 system on this biomarker for both LP and MLPs. Notice an improvement on LP over MLPs for GM00593 (p = *0.0259*), GM00958 (p = *0.0330*) fibroblasts, and GM01259 (p = *0.0291*) fibroblasts, when CRISPR/nCas9 and Donor AAVS1:GALNS plasmids were transfected. Fibroblasts were transfected either with the Donor AAVS1:GALNS plasmid (Donor) or the CRISPR/nCas9-Donor AAVS1:GALNS plasmids (CRISPR/nCas9:Donor). (**B**) Left histograms to correspond to representative findings of MFI for LysoTracker Deep Red observed on MPS IVA after CRISPR/nCas9:Donor AAVS1 treatment. The mean of relative MFI for each MPS IVA fibroblast to untreated cells (n = 4) is shown on the right. The horizontal dotted line represents the relative MFI of wild-type fibroblasts. Note that LP led a better improvement for GM00593 (p = *0.0284*) and GM01259 (p = *0.0161*) fibroblasts compared MLPs; while an opposite behavior was achieved for GM01361 fibroblasts (p = *0.0461*). Fibroblasts were transfected either with the Donor AAVS1:GALNS plasmid (Donor) or the CRISPR/nCas9-Donor AAVS1:GALNS plasmids (CRISPR/nCas9:Donor). (**C**) Representative fluorescent images for LysoTracker Deep Red staining*.* Red fluorescence corresponds to lysosomal mass, while the blue signal to the nucleus. *UT* untreated, *DG* donor AAVS1:GALNS, *CD* CRISPR/Cas9 plus Donor AAVS1:GALNS. *p ≤ 0.05, **p ≤ 0.005, ***p ≤ 0.001, ****p ≤ *0.0001*. Two-tailed Student's t-test.

We evaluated the impact of the CRISPR/nCas9 treatment on the total GAGs and lysosomal mass, the major biomarkers of MPS IVA^11,35^. We found a normalization in GAGs levels when CRISPR/nCas9 system was delivered with LP to GM00593 and GM01361 fibroblasts. At the same time, a significant reduction was observed with MLPs (Fig. 4A). Although we failed to observe a normalization in the GAGs levels for GM00958 and GM01259 fibroblasts, treatment with both LP and MLPs led to a significant decrease in such levels (Fig. 4A).

In line with these findings, compared to wild-type, we found a significant lysosomal mass increase in all the untreated fibroblasts (Fig. 4B). CRISPR/nCas9-based genome editing on GM00593 and GM01361 fibroblasts, through either LP or MLPs, reduced the lysosomal mass to wild-type levels (Fig. 4B). Moreover, GM00593 fibroblasts showed a significant decrease (~ 8% respect untreated, *p* = *0.001*) after treatment with only the MLPs and Donor AAVS1:GALNS plasmid, suggesting a residual effect of GALNS activity. This agrees well with the slight increase observed in enzyme activity (Fig. 3). Similar to the GAGs results, edited GM01259 fibroblasts showed a decrease in lysosomal mass for both LP (20%, respect to untreated cells, *p* = *0.0143*) and MLPs (5%, respect to untreated cells, *p* = *0.0204*), which were significantly different to the WT levels (LP: *p* = *0.0423*; MLPs: *p* = *0.0010*). In contrast, no significant changes were observed in lysosomal mass for GM00958 fibroblasts compared to untreated cells after the CRISPR/nCas9-based genome edition, suggesting that 13% of GALNS activity was insufficient to reduce the lysosomal mass, despite the slight decrease in GAGs levels (Fig. 4B). Similar findings were observed in lysosomal mass analyzed via fluorescence microscopy assays for all the evaluated fibroblasts (Fig. 4C). Overall, these observations support the closest relationship between GALNS levels and the recovery of the lysosomal function and highlight the need to develop strategies to increase GALNS enzyme activity levels above 13% of wild-type.

### Partial restoration of β-hexosaminidase activity and mitochondrial-dependent oxidative stress (mtROS) is observed after CRISPR/nCas9-based genome editing

**Figure 5:**
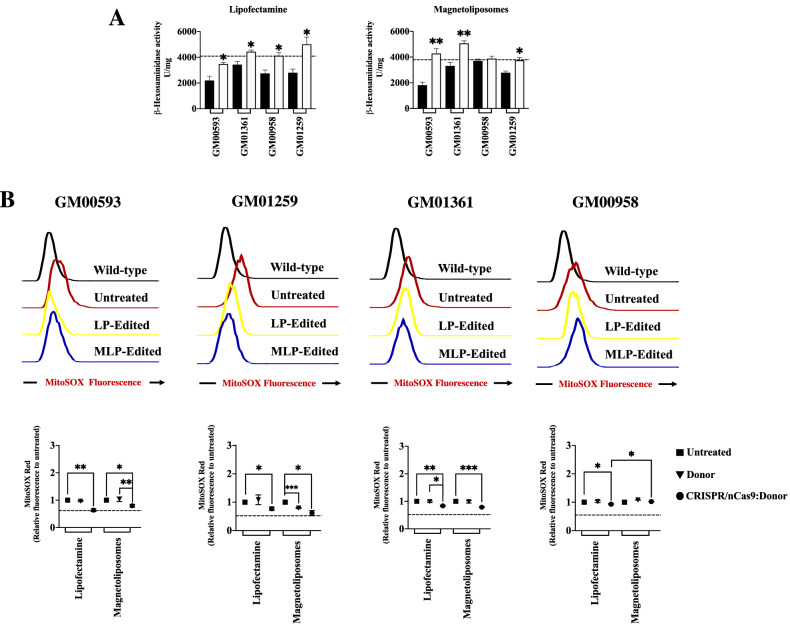
Restoration of lysosome flux and mtROS. (**A**) Recovery of total β-hexosaminidase enzyme activity for LP (Left) and MLPs (Right) treated cells (n = 4). The horizontal dotted line represents the normal enzyme values on the wild-type fibroblasts. (**B**) The upper panel shows the representative histogram of relative MitoSOX red MFI for CRISPR/nCas9:Donor AAVS1 treatment. The bottom panel is the mean for each MPS IVA fibroblast obtained from experiments like the upper one (n = 4). GM00958 fibroblasts were responsive when LP was used for the CRISPR/nCas9 system delivery. Fibroblasts were transfected either with the Donor AAVS1:GALNS plasmid (Donor) or the CRISPR/nCas9-Donor AAVS1:GALNS plasmids (CRISPR/nCas9:Donor). *p ≤ 0.05, **p ≤ 0.005, ***p ≤ *0.001*. Two-tailed Student's t-test.

As in any other LSD, the main cause of the disease pathology in MPS IVA may be associated to the build-up of partially degraded substrates (i.e. KS and QS). Nevertheless, this build-up does not fully explain the pathophysiology of the disease, and it has been reported that the abnormal lysosomal function may alter other lysosomal enzymes, such as β-hexosaminidases (Hex)^36,37^, as well as lead to an increase in cellular oxidative stress^3,4,38^. In this sense, we observed a lower total Hex activity in all the untreated MPS IVA fibroblasts (fold change: 0.44–0.84) compared to WT levels (4089 ± 648 U/mg) (Fig. 5A). The lowest Hex activity was observed for GM00593 fibroblasts (1815 ± 235.6 U/mg), while GM01361 fibroblasts (3314 ± 267 U/mg) were affected the least. A significant increase in Hex levels was observed for all the MPS IVA fibroblasts after treatment with CRISPR/nCas9 using LP (Fig. 5A). Hex levels were also significantly increased for GM00593 and GM01361 fibroblasts using MLPs, while a significant decrease was observed for GM00958 and GM01259 fibroblasts (Fig. 5A).

As mentioned above, an increase in oxidative stress has been established as an important pathological state in MPS IVA patients^3,4^. Noteworthy, the current ERT fails to recover them from such state^4^. We observed that MPS IVA fibroblasts have increased mtROS levels, which were reduced after CRISPR/nCas9-based treatment (Fig. 5B). mtROS levels were normalized on GM00593 fibroblasts treated with LP, while treatment with MLPs allowed a 21% reduction compared to untreated cells (*p* = *0.0177*). An opposite behavior was observed for GM01259 fibroblasts, in which MLPs treatment led to the restoration of mtROS to wild-type levels. At the same time, a significant reduction of 23% was observed with LP compared to untreated cells (*p* = *0.0041*). GM01361 fibroblasts showed a significant improvement with both LP- (17%, *p* = *0.0015*), and MLPs-transfection (22%, *p* = *0.0006*), compared with absence of treatment (Fig. 5B). Finally, GM00958 fibroblasts were the lowest responsive cells, with a slight decrease (7%) for the LP treatment (*p* = *0.0498*). No significant changes in mtROS were observed when only the Donor AAVS1:GALNS was transfected with either LP or MLPs (Fig. 5B), suggesting that phenotype rescue was due to the CRISPR/nCas9-based genome editing and not to the eventual episomal persistence of the Donor AAVS1:GALNS. These results show a consistent recovery of two critical biomarkers in a GALNS increased activity-dependent manner and continue supporting the suitability of MLPs as non-viral vectors for the delivery of CRISPR/nCas9 systems.

### Stable CRISPR/nCas9-mediated genome editing fails to alter MPS IVA fibroblasts' Nitric oxide (NO)-related inflammatory/oxidative profile

**Figure 6:**
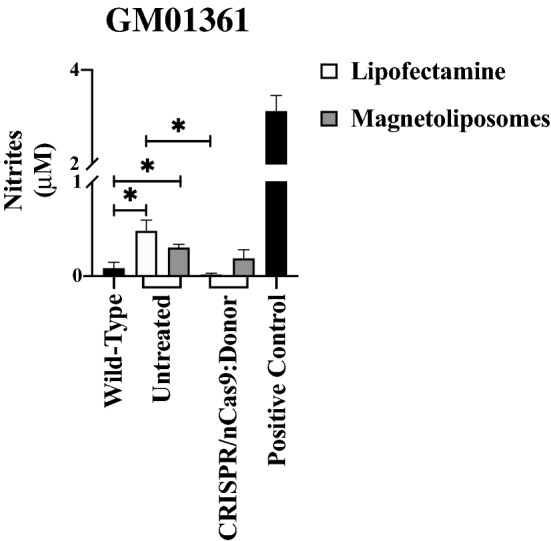
Effect of MLPs-attached CRISPR/nCas9 on the global profile of NO-derived species in MPS IVA fibroblasts. Mean of 4 experiments for GM01361 fibroblasts after transfection with CRISPR/nCas9-Donor AAVS1:GALNS plasmids (CRISPR/nCas9:Donor) using LP and MLPs as the delivery system. Note that untreated cells showed a significant increase in nitrites amounts compared to wild-type. LPS was used as positive control on wild-type cells. *p ≤ *0.05*. Two-tailed Student's t-test.

Given that CRISPR/nCas9 is a bacterial-derived genome editing tool^16,39^ and that the MLPs contain iron oxide^31^, we decided to test the presence of NO_2_ in cells treated with LP- or MLPs-transfection. Interestingly, we did not find any substantial difference between wild-type and GM00593, GM00958, and GM01259 fibroblasts before or after the CRISPR/nCas9 genome editing (Supplementary Fig. 5). However, untreated GM01361 fibroblasts showed a significant NO_2_ increase compared to wild-type cells (0.481 ± 0.11 vs. 0.084 ± 0.06 µM; *p* = *0.0380*), suggesting a basal NO-related proinflammatory phenotype in these cells (Fig. 6). Noteworthy, after treatment with the CRISPR/nCas9-based genome editing tool, a significant 33-fold reduction in NO_2_ levels was observed with LP transfection (*p* = *0.0153*); however, this was not the case for the MLPs-transfection (Fig. 6).

## Discussion

Iron oxide-based nanoparticles, particularly magnetite, have attracted significant attention over the past two decades for developing biomedical applications mainly because iron is a common element that humans can metabolize^40,41^. In this regard, MNPs-based formulations have been approved by the Food and Drug Administration (FDA), mainly for magnetic resonance imaging purposes^42,43^ and a few more for addressing Iron metabolic disorders such as Iron deficiency anemia^44^. In this study, we assessed MLPs (which are based on magnetite encapsulated into liposomes) as potential in vitro delivery vehicles for a CRISPR/nCas9 genome editing designed to treat MPS IVA. Previously, a nanovehicle was developed based on MNPs covered with a patchy silver shell to which a pH-responsive polymer was conjugated along with the cell-penetrating peptide Buforin II attached to the core (MNPs@Ag-pD/BUF-II). This nanovehicle was proven effective to load plasmids and escape from the endolysosomal pathway without decreasing cell viability of several in vitro models^31^.

The surface composition of non-viral vectors is crucial for biodistribution^45^. For example, iron-based nanoparticles exhibiting zeta potential ranging − 30 to + 11.57 mV and sizes ranging from 135 to 221 nm have shown an increased internalization on in vitro assays, and with preference liver storage has been found in the liver for in vivo approaches^46,47^. Since our MLPs showed a hydrodynamic diameter of 247 nm and Z-potential of − 38.9 ± 2.4 mV (Fig. 1B,C), which were consistent with our previous results^31^, we believe that including liver-specific promoters to our current plasmids constructs, could be helpful to use the liver as a transgene factory producer. Classical GT using AAV has shown the suitability of the liver for transgene expression^48^.

Although the central goal of this work was not to characterize in depth the process behind MLPs uptake, we performed preliminary experiments to determine the possible mechanism of internalization. As expected, after 4 h of incubation, only fibroblasts incubated at 37 °C but not at 4 °C were able to internalize MNPs@Ag-pD/BUF-II (Fig. 1E), strongly suggesting endocytosis as the main uptake mechanism^49,50^. Additional experiments will be needed to elucidate if classical clathrin- or caveolin-related pathways might be involved in the MNPs@Ag-pD/BUF-II internalization, as described for similar nanostructures^51–53^.

Although iron oxide-based nanoparticles may have low cytotoxicity for several cell lines^31,54^, MPS IVA fibroblasts could be more susceptible due to the deregulation of caspase 3/7-dependent apoptotic pathways^55^. To elucidate this premise on MPS IVA patients’ fibroblasts, we evaluated the cytotoxic and proapoptotic effects of the MLPs. After incubation with MLPs, less than 10% of apoptotic cells were observed (Fig. 1F), which aligns with previous reports on iron-based nanoparticles^31,56,57^.

Regarding the transfection efficiency, it was observed that MLPs were more efficient than LP in all the MPS IVA fibroblasts, with an increase between 1.5- and 21.1-fold, respectively (Fig. 1I). Interestingly, this increases in transfection efficiency with MLPs, compared to LP, led to no significant changes in enzyme activity and phenotype. For instance, although GM00958 and GM01259 fibroblasts showed the highest transfection levels with MLPs, they showed the lowest responses in some of the evaluated MPS IVA biomarkers.

After translation, GALNS undergoes post-translational modification in the endoplasmic reticulum (ER)-Golgi apparatus (GA) pathway to attach mannose motifs in the Asn204/Asn423 residues and phosphorylate them, respectively^58^, which are crucial for sorting of lysosomal proteins^59,60^. In this regard, structural analysis of intracellular organelles of MPS IVA fibroblasts has revealed changes in the number, length, and width on the GA but not on the ER^61^. Consequently, we hypothesized a partially impaired processing of the overexpressing GALNS enzyme in the GA after Donor AAVS1:GALNS insertion in the *AAVS1* locus. Under our experimental conditions, we cannot exclude potential ER stress. In fact, it has been reported that overexpressing lysosomal enzymes contributes to unwanted effects on ER, including the expression of unfolding protein response-related genes^62,63^. Further experiments will be conducted in future works to test our hypothesis.

Enzyme activity levels ranged from 31 to 62% of the WT levels in GM00593 fibroblasts and 29.7% and 21% of the WT levels for GM01361 fibroblasts after LP and MLPs treatment, respectively. These GALNS levels were enough to observe a positive response in almost all the evaluated biomarkers. These results agree with our previous findings using the same CRISPR/nCas9 system in MPS IVA fibroblasts transfected with lipofectamine^11^. In contrast, GM01259 fibroblasts showed a slightly lower recovery than GM00593 and GM01361 fibroblasts, while GM00958 showed the lowest response. A similar observation has been previously reported for GM00958 fibroblasts for several treatments, including PC^64^ and lentiviral-based GT^35^. As proposed earlier, our results strongly support the notion of a mutation-dependent response on MPSIVA fibroblasts^35,64^.

Oxidative stress plays an essential role in the pathophysiology of MPS IVA, which cannot be ameliorated with ERT^4^. In this sense, an increase in the mtROS levels was observed, compared to wild-type levels, in all the evaluated MPS IVA fibroblasts (Fig. 5B). After the CRISPR/nCas9-based genome edition, a phenotype rescue was observed for most cells using both LP and MLPs as delivery vectors. In general, an increase in mtROS levels can be attributed to several factors, such as the direct interaction between the mitochondria and lysosome and the defective autophagy efflux, which is crucial to renewing the mitochondria network in a process known as mitophagy^65^. In agreement with the lysosomal mass and mtROS results, we hypothesize that the CRISPR/nCas9-based treatment could restore the autophagy flux. This relationship between improvement of lysosome and autophagy after GALNS increase has been reported for a lentiviral-based GT^35^. Further experiments will be therefore dedicated to test this rationale.

Notably, the GM00958 fibroblasts failed to improve lysosomal mass after treatment with CRISPR/nCas9. However, a slight reduction in mtROS was observed after treatment with LP, despite the low GALNS activity achieved (13% of WT levels). Although these cells have not been tested for mtROS previously using other GT approaches, it was recently described that GM00958 fibroblasts remain unresponsive after treatment with a lentiviral vector carrying GALNS, with or without SUMF1 co-transduction^35,66^. Since we observed an improvement in the enzymatic activity in these fibroblasts after CRISPR/nCas9-based genome editing, we hypothesize that co-delivering an expression cassette containing SUMF1 into the Donor AAVS1:GALNS might be able to elicit a more pronounced phenotype rescue in GM00958 fibroblasts.

Overall, based on the biomarkers evaluated after CRISPR/nCas9-based genome editing, we observed GM00593 and GM01361 fibroblasts as the most responsive cells for both LP- and MLPs-treatments (Supplementary Table 1). Interestingly, these cells have two composed mutations represented by p.R386C/p.F285del and p.R61W/W405_T406del^67^, which are related to a severe phenotype in MPS IVA patients^67,68^. Moreover, for the least responsive GM00958 and GM01259 fibroblasts, previous works have reported on the presence of point mutations p.A393S and p.R94C/A393S, respectively^67^. These mutations were predicted as benign or neutral and associated with a less severe phenotype than the previous one^67^. Notably, GM02159 fibroblasts showed a residual GALNS activity that approached 40% of WT levels (Fig. 3); however, previous reports describe a significant alteration of classical disease biomarkers^67^. Overall, these findings suggest that CRISPR/nCas9-based GT could potentially have an enhancer response for patients with severe phenotypes compared with the attenuated ones.

Although CRISPR/nCas9-based GT is a promising therapeutic strategy due to the delivery of a normal version of the *GALNS* gene into a safe harbor that should be enough to recover the enzyme function, the results of the present study showed that this premise could not be entirely accurate. Even though we previously demonstrated that the Donor AAVS1:GALNS is inserted on the *AAVS1* locus^11^, different epigenetic backgrounds could be detrimentally impacting the outcome of the mutation evaluated, as evidenced by the incomplete phenotype recovery in some of the models^69^. In fact, for the *GALNS* gene, Tomatsu et al., observed fundamental changes in the methylation pattern^70^. We have also identified changes in methylation and histone acetylation with apparent differences between mutations on the same cell models tested in this study^71^. Consequently, we cannot discharge a potentially unwanted impact (i.e., partial methylation-dependent silencing) of our *knock-in* approach on this altered epigenetic pattern.

## Conclusion

In this study, we have expanded our previous findings related to the potential use of a CRISPR/nCas9-based genome edition system to four in vitro models of MPS IVA containing different mutations using a non-viral vector based on MLPs for the delivery of the CRISPR/nCas9 system. Under our experimental conditions, we found a significant increase in the GALNS activity for all the MPS IVA fibroblasts evaluated. The results also demonstrated the reduction in lysosomal mass, GAGs, and mtROS after treatment with CRISPR/nCas9 using both the conventional LP and the novel MLPs. Recovery on the β-hexosaminidase activity was also observed after transfection of the CRISPR/nCas9 system. These results confirmed the potential of CRISPR/nCas9 as a promising strategy for MPS IVA treatment when used in conjunction with a non-viral vector. Noteworthy, we demonstrated the importance of including different genetic backgrounds during the evaluation of therapeutic strategies since this is critical for the eventual success of the therapy. Experiments in MPS IVA mice are underway to verify the therapeutic potential of this approach in vivo and consequently devise a much more comprehensive route for clinical translation.

## Materials and methods

### Synthesis of cell-penetrating bioconjugates and liposomes

Magnetic nanoparticles (MNPs) were prepared by a chemical coprecipitation of FeCl_2_ and FeCl_3_ (2:1 molar ratio), as described previously^31^. A silver shell was then formed on the surface of MNPs by a redox reaction in the presence of Ag ions. A solution containing 2 mg/mL MNPs and 1 mM AgNO_3_ was stirred (500 rpm) for 10 min. An equivalent volume of honey solution was subsequently added and stirred for (500 rpm) 10 min. Finally, the reduction of Ag^+^ ions was initiated by increasing pH to 8.5 with 5 M NaOH added dropwise. The solution was stirred (500 rpm) at room temperature for one hour.

A Hofmann elimination reaction achieved the conjugation of the pH-responsive polymer poly(2-(dimethylamino)ethyl methacrylate) (pDMAEMA, Sigma-Aldrich, USA). For this, 2 mg/mL of the core–shell MNPs:Ag were sonicated, and then 1 mM HCl was added until the pH decreased to 3. MNPs were dispersed in type I water (50 mL, 2 mg/mL) and pDMAEMA (10 mL, 2 mg/mL) was slowly added resulting in MNPs:Ag-pDMAEMA. Later, they were functionalized with 3-aminopropyl)triethoxysilane (APTES, Sigma-Aldrich, USA), followed by conjugation of amino PEG12 propionic acid (PEG12, Sigma-Aldrich, USA). Finally, Buforin II was immobilized on the resulting MNPs:Ag-pDMAEMA/PEG12 conjugates to form the MNPs:Ag-pDMAEMA/PEG12-BUF-II nanobioconjugates, hereafter called MNPs@Ag-pD/BUF-II. Additional details can be consulted elsewhere^31^.

Liposomes were synthesized by the lipid bilayer hydration method^72^. In brief, soy lecithin was dissolved in chloroform (10 mg/mL). The solution was evaporated in a rotary evaporator (Hei-VAP Core, Heidolph, Germany) under vacuum at 45 °C for one hour. The film was hydrated with PBS (1X) in the rotary evaporator at 55 °C for one hour. Finally, the solution was filtered (0.22 μm) and stored at 4 °C until further use.

### Physical characterization of nanobioconjugates

Hydrodynamic size and Z-potential were measured by using dynamic light scattering (DLS) in a Zeta-Sizer Nano-ZS (Malvern Panalytical, Malvern, UK) according to our previous protocols^31^. For loading DNA capacity, we used the AIO-mCherry plasmid (Addgene: #74120). DNA loading was calculated as reported by Ramirez et al.^31^ and defined as the maximum amount of plasmid DNA loaded per μg of MNPs@Ag-pD/BUF-II. Briefly, 250 μg of MNPs@Ag-pD/BUF-II were incubated with plasmid DNA ranging between 0 and 3000 ng.

### Protection against *DpnI* digestion

For the *Dpn*I assay, we used one plasmid CRISPR/nCas9 (pDNA) containing two sgRNA engineered in the *AAVS1* locus^11^. Initially, 28 µg MNPs@Ag-pD/BUF-II suspended in 30 µL PBS 1X pH 8.0 were precipitated using a magnetic field aided by a PureProteome Magnetic Stands (Merck Millipore, USA) for 10 min. The supernatant was gently removed, and 1 µg of plasmid previously diluted in Cut Smart buffer 1X (CSB, New England Biolabs, USA) was mixed with the precipitated MNPs@Ag-pD/BUF-II. The solution was vigorously mixed by pipetting up and down, and it was incubated for 10 min at RT/130 rpm. Later, MNPs@Ag-pD/BUF-II:pDNA complexes were either left alone or mixed with 0.1 mg/mL liposomes dissolved in CSB at a 1:1 ratio. An additional incubation for 10 min was carried out at room temperature and 20 rpm to obtain MLPs. Finally, the MLPs carrying out the plasmids were incubated with 20U *Dpn*I for 30 min at 37 °C, according to previous reports^73,74^. Uncoupled pDNA (naked), as well as untreated one, were included as controls.

The samples were submitted at 80 °C for 10 min. Later, 80 µL phenol:chloroform:isoamyl alcohol 25:24:1 were added and incubated for 10 min at room temperature. After centrifugation at 10,000 rpm for 5 min, ~ 20µL aqueous phase was loaded on agarose gel 1% p/v stained with 0.5 μg/mL ethidium bromide and run at 100 V for 1 h.

### Mammalian cell culture

GM00593, GM01361, GM00958, and GM01259 fibroblasts were purchased from the Coriell Institute repository (New Jersey, USA). Specific characteristics of each cell are registered in Supplementary Table 1. Wild-type fibroblasts were isolated from apparently healthy men through informed consent. The cells were maintained in Dulbecco's Modified Eagle Medium (DMEM, Biowest; USA) supplemented with not inactivated 15% Fetal Bovine Serum (FBS, Biowest; USA), 100 U/mL penicillin, and 100 µg/mL streptomycin (Gibco, USA). The assays were carried out using cells in passages between 3 and 7. All the experiments were approved by the Research and Ethics Committee of the Faculty of Science at Pontificia Universidad Javeriana (Minutes 06, 2018).

### CRISPR/nCas9 and donor AAVS1:GALNS plasmids

We used a recently validated CRISPR/nCas9 system for this study, which allows the efficient homologous recombination of an expression cassette containing a functional version of the human *GALNS* gene^11^. Briefly, two sgRNA were engineered to address nCas9 at the adeno-associated virus integration site (*AAVS1*) locus (NCBI Accession: S51329.1) and later cloned into AIO-mCherry plasmid (Addgene: #74120), which carries the CRISPR/nCas9 system, using the golden gate strategy, as described previously^17^. No detectable sequence changes on the Top 10 *Off-targets* were identified by Sanger sequencing^11^. Donor AAVS1:GALNS contains an encoding wild-type sequence of the human *GALNS* gene (NCBI Accession: NM_000512) (Supplementary Fig. 1). We encourage readers to review Leal & Alméciga, 2022, for details^11^.

### Cytotoxic effect

To determine the cytotoxic effect of liposomes and MNPs@Ag-pD/BUF-II, we performed two screenings using both MTT (3-(4,5-dimethylthiazol-2-yl)-2,5-diphenyltetrazolium bromide; Sigma-Aldrich, USA) and an LDH Cytotoxicity Detection Kit (Lactate dehydrogenase release, Roche, Germany). Briefly, 10,000 cells/well were seeded on 96-wells plates (TPP; Switzerland) 24 h before assays. Liposomes or MNPs@Ag-pD/BUF-II were incubated with the cells in concentrations ranging from 30 to 500 μg/mL and 12.5–100 μg/mL, respectively, for 48 h. The plates were read in a microplate reader (Biochrom Anthos 2020, UK) at 562/630 nm and 490/630 nm for MTT and LDH, respectively.

The results showed no observable cytotoxic effects at 0.1 mg/mL liposomes and 50 μg/mL MNPs@Ag-pD/BUF-II (data not shown). Moreover, to evaluate the potential synergic cytotoxic effect of the MLPs, we performed the same screening by using 25 μg/mL/0.05 mg/mL MNPs@Ag-pD/BUF-II:liposome ratio. The cells were also visualized under Differential Interference Contrast (DIC) with an Axio Observer Z1 microscope (ZEISS, Birkerød, Denmark) to identify morphology changes after treatment. The images were analyzed with the ImageJ software^75^.

### Apoptosis assay

To evaluate whether the MLPs have a proapoptotic effect, we used Alexa Fluor 488 Annexin/Dead Cell Apoptosis Kit (Thermo Fisher Scientific, USA). Briefly, 30,000 cells/well were grown on 12-well plates (TPP, Switzerland) for 24 h. Next, the MLPs 25 μg/mL/0.05 mg/mL nanobioconjugate/liposome ratio were added to each well, and the cells were incubated for 48 h. After the incubation, the cells were harvested by trypsinization, washed twice with warmed phosphate buffer saline 1X (PBS 1X), and incubated with Annexin V and Propidium Iodide (PI) for 5 min at room temperature. Finally, the cells were analyzed by flow cytometry in a BD FACSAria III Cell Sorter. At least 50,000 events were registered on each assay, and the data were analyzed with the FlowJo software. As a positive control, we used fibroblasts incubated with doxorubicin 2.5 μM by 72 h, as reported previously^76^. Phosphatidylserine externalization was used as a marker for early apoptosis by attaching Annexin V-Alexa Fluor 488 (*Ex/Em*: 488/530 nm), while necrotic cells were identified with PI (*Ex/Em*: 488/695 nm) staining. Double positive cells (Annexin^+^/PI^+^) were considered late apoptotic cells.

### Internalization assay

30,000 cells/well PolyD-Lysine-treated coverslip seeded in 12-well plates (TPP; Switzerland) were incubated with MLPs containing Rhodamine-labeled MNPs@Ag-pD/BUF-II (Rho-MLPs) at 4 °C, and 37 °C for 4 h. Later, the media culture was removed, and the cells were washed twice with cold PBS 1X and fixed with 4% paraformaldehyde in PBS for 15 min. Three additional washes were performed with 1X PBS. The coverslips were put on slides containing a fluoroshield mounting medium with DAPI (Abcam; UK). The cells were observed with an Axio Observer Z1 microscope (ZEISS, Birkerød, Denmark) with a Filter set 109 HE LED (E), containing beam splitter TBS 405 + 575 and an emission filter TBP 425/29 + 632/100; this configuration collected DAPI and Rhodamine, respectively. Images were processed with the ImageJ software^75^.

### Transfection assisted by lipofectamine

We used Lipofectamine 3000 (LP, Thermo Fisher Scientific, USA) as a standard transfection method following previously standardized protocol^11^. Briefly, 30,000 cells/well were transfected with 1 µg pDNA (0.5 µg pDNA CRISPR/nCas9 and/or 0.5 µg pDNA Donor AAVS1:GALNS) for 24 h in 12-well plates (TPP, Switzerland). After the incubation, the culture media was replenished by fresh one.

### Transfection assisted by magnetoliposomes

As for the LP experiments, 30,000 cells/well were used in experiments with magnetoliposomes. CRISPR/nCas9 and/or Donor AAVS1:GALNS plasmids were complexed to NP as reported previously^31^. A total of 25 µg of MNPs@Ag-pD/BUF-II were used for all the experiments containing up to 0.88 µg pDNA (~ 0.44 µg pDNA CRISPR/nCas9 and/or ~ 0.44 µg pDNA Donor AAVS1:GALNS). pDNA coupling and MLPs synthesis were performed as described for the *Dpn*I assay. Finally, the MLPs carrying out the plasmids were added dropwise to each well for 4 h at 37 °C/5%CO_2_. At that point, the culture media was removed and replenished by fresh one. All the experiments regarding genome editing (LP and MLPs) were completed on cells treated for 1 month to determine the long-term impact of the edition on MPS IVA fibroblasts. Wild-type fibroblasts were used as normal control. Naked DNA was included as a control in all the experiments, with a non-significant effect on any determination (data not shown).

### RT-qPCR

One-month post-transfection with LP or MLPs, the fibroblasts underwent RNA isolation using a Monarch Total RNA Miniprep Kit (New England Biolabs; USA). RNA quality was checked by running an agarose gel under denaturing conditions and quantified by NanoDrop 1000 spectrophotometer (Thermo Fisher Scientific, USA). 0.5 µg of total RNA was retrotranscribed through High-Capacity cDNA Reverse Transcription Kit (Applied Biosystems; USA). The resulting cDNA was used to run an absolute quantitative real-time PCR (RT-qPCR) to determine the expression of *GALNS* by using the TaqMan probe Hs00975732_m1-FAM (Applied Biosystem; USA). A standard curve using Donor AAVS1:GALNS was performed to determine the number of copies/µL.

### GALNS enzyme assay

Total protein from fibroblasts was extracted using deoxycholate 1% plus phenylmethylsulfonyl fluoride 1 mM (PMSF, Sigma-Aldrich, USA). The protein concentration was determined using a BCA Protein Assay Kit (Thermo Fisher Scientific, USA). The GALNS enzyme assay was conducted as described by Leal and Alméciga^11^. Briefly, 10 µL of protein were mixed with 20 µL/2 mM 4-methylumbelliferyl-β-d-galactopyranoside-6-sulfate (4-MU-Gal-6S, Toronto Chemicals Research, North York, ON, Canada) during 17 h at 37 °C. Later, 2 µL of β-galactosidase (10 mg/mL; Sigma-Aldrich, USA) was added to the reaction for 4 h. Next, 150 µL of glycine-carbonate pH 9.8 were used to stop the reaction. The samples were transferred to a 96-wells black flat-bottom (Corning, USA) and read in a fluorometer Twinkle LB970 (*Ex/Em*: 365/450 nm; Berthold Technologies). To determine the fluorescence of 1 nmol, a standard curve of 4-methylumbelliferone (Sigma-Aldrich; USA). Specific GALNS activity was expressed as U/mg of total protein.

### Glycosaminoglycans quantification

Total GAGs were determined on extracellular media as described recently^11^. One-week post-treatment, 300 µL of supernatant were collected every 4 days and stored at − 20 °C for up to one month. At that time, an aliquot of 50 µL were incubated with 275 µL of 1% p/v 1,9-dimethyl methylene blue (DMB) pH 3.3–2 M Tris-Base for at least 1 min and for up to 5 min. A spectrophotometer BioSpec-1601 (Shimadzu, USA) was used to read samples at 520 nm. The results were compared against a standard curve of chondroitin sulfate A (Sigma-Aldrich. USA).

### Lysosomal mass determination

Lysosomal determination was evaluated by two approaches: epifluorescence microscopy and flow cytometry. In the first case, the fibroblasts were seeded on coverslips treated with 50 µg/mL PolyD-Lysine (Gibco, USA) before LP- or MLPs-transfection procedure. After one month of treatment, the cells were fixed with paraformaldehyde 4% and stained using LysoTracker Deep Red (L12492; Thermo Fisher Scientific, USA) as described previously^11,35^. Later, the cells were also stained with DAPI (Fluoroshield mounting medium, Abcam, UK) to observe the nucleus. Coverslips cells were placed on slides and then visualized using an Axio Observer Z1 microscope (ZEISS, Birkerød, Denmark) with a Filter set 109 HE LED (E), containing beam splitter TBS 405 + 575 and an emission filter TBP 425/29 + 632/100. This configuration collected the DAPI and Lysotracker signals, respectively. The images were processed with the ImageJ software^75^. Regarding flow cytometry, a BD FACSAria™ III Cell Sorter (*Ex/Em*: 647/668 nm) was used to acquire at least 50,000 events following the protocol described by Leal & Alméciga, 2022^11^. Propidium iodide 1 mg/mL (Sigma-Aldrich; USA) was used to identify viable cells. Only the mean fluorescence intensity (MFI) from singlets was included for the analysis on FlowJo software.

### Mitochondrial-derived reactive oxidative species (mtROS)

The MitoSOX Red indicator (Thermo Fisher Scientific, USA) was used to evaluate mtROS in cells treated for one month. A cell monolayer was incubated with 2.5 µM MitoSOX in Hank’s Balanced Salt Solution (1X) for 15 min. At least 10,000 events were recorded in a Cytek Aurora CS cytometer using PE—Red (*Ex/Em*: 510/580 nm) channel. 100 µM CoCl_2_ (Sigma-Aldrich, USA) was used as a positive control according to previous reports^11^. Treatment with CoCl_2_ failed to promote cell death (data no show).

### Hexosaminidase enzyme assay

After LP- or MLPs-transfection, the cells were lysed with deoxycholate 1% plus 1 mM PMSF. Later, 50 µL of the sample were incubated with 20µL/32 mM 4-MU-NAG (4-Methylumbelliferyl *N*-acetyl-β-d-glucosaminide, Sigma-Aldrich, USA) for 20 min at 37 °C^77–79^. 150 µL 0.2 M glycine pH 10.8 were used as a stop buffer solution. Samples were read and analyzed as described for the GALNS enzyme assay.

### Griess determination

The presence of nitric oxide-derived species (NO) was determined by a test based on the Griess reagent (Sigma-Aldrich, USA)^80^. Briefly, 100 µL of supernatant collected from untreated or treated cells were incubated with 100 µL of the Griess reagent for 15 min at room temperature. 1 µM lipopolysaccharide *Escherichia coli* O111:B4 (Sigma-Aldrich, USA) was used as a positive control. Samples were read in a microplate reader (Biochrom Anthos 2020, UK) at 562/630 nm. The results were extrapolated to a nitrite standard curve, which was performed using NaNO_2_ (Sigma-Aldrich, USA).

### Statistical analysis

The data was processed in GraphPad Prism version 8.0.0 for Mac (GraphPad Software, San Diego, California, USA). Normal distribution and homoscedasticity were evaluated through Shapiro–Wilk Test and Leve Test, respectively. Mean comparison between groups was addressed with a t-student test, a Mann–Whitney U test, or an ANOVA, according to the Shapiro–Wilk test findings. The experiments were performed in at least three independent experiments in triplicate. All the data is presented as mean ± standard error (SE). *p* < *0.05* was considered statistically significant.

## Supplementary Information

Supplementary Information.

## Data Availability

The datasets generated during and/or analyzed during the current study are available from the corresponding author on reasonable request.
