# Supplementary material for: Delivery and assessment of a CRISPR/nCas9-based genome editing system on in vitro models of mucopolysaccharidoses IVA assisted by magnetite-based nanoparticles
Source: Sci Rep. 2022 Sep 3;12:15045. doi: 10.1038/s41598-022-19407-x (PMC9440901; doi:10.1038/s41598-022-19407-x)
Supplement: Supplementary file 1 — Supplementary Information. [file 41598_2022_19407_MOESM1_ESM.pdf]

Delivery and assessment of a CRISPR/nCas9-based genome editing system on *in vitro* models of mucopolysaccharidoses IVA assisted by magnetite-based nanoparticles

Andrés Felipe Leal[1], Javier Cifuentes[2], Carlos Emilio Torres Garay[2], Diego Suárez[1], Valentina Quezada[2], Saúl Camilo Gómez[2], Juan C. Cruz[2], Luis H. Reyes[3], Angela Johanna Espejo-Mojica[1], Carlos Javier Alméciga-Díaz[1*]

[1]Institute for the Study of Inborn Errors of Metabolism, Faculty of Science, Pontificia Universidad Javeriana, Bogotá D.C., 110231.

[2]Department of Biomedical Engineering, Universidad de los Andes, Bogotá 111711, Colombia

[3]Grupo de Diseño de Productos y Procesos (GDPP), Department of Chemical and Food Engineering, Universidad de los Andes, Bogotá 111711, Colombia

***Corresponding author:**

Carlos J. Alméciga-Díaz, BPharm, Ph.D., Instituto de Errores Innatos del Metabolismo, Pontificia Universidad Javeriana, Cra. 7 No. 43-82 Building 54, Room 305A. Bogotá D.C., 110231, Colombia. Tel: +57-1 3208320 Ext 4140; Fax: +57-1 3208320 Ext 4099; E-mail: cjalmeciga@javeriana.edu.co.

Content

Supplementary Table 1. Characteristics of MPS IVA fibroblasts used in this study and their global response to the CRISPR/nCas9-based genome editing 3

Supplementary Figure 1. CRISPR/nCas9 and Donor AAVS1:GALNS vectors 4

Supplementary Figure 2. Unprocessed *DpnI* digestion gel.. 5

Supplementary Figure 3. Cytotoxicity assays for MPS IVA fibroblasts incubated with MLPs.. 6

Supplementary Figure 4. Extracellular release of functional GALNS enzyme on GM00593 fibroblasts... 7

Supplementary Figure 5. Nitrites determination assay on MPS IVA fibroblasts after CRISPR/nCas9 treatment... 8

Supplementary References... 9

Supplementary Table 1. Genetic background of MPS IVA cells used in this study and overall response upon CRISPR/nCas9 system using Lipofectamine (LP) or magnetoliposomes (MLPs) as a delivery strategy. Bold numbers represent a non-significant change after treatment. **Trans:** Transfection strategy. **GALNS Act:** Intracellular GALNS activity. **GAGs:** Glycosaminoglycans. **LM:** Lysosomal mass. **β-Hexo**: Total intracellular β-Hexosaminidase activity. **F:** Female. **M:** Male. **FC:** Fold-Change. *Obtained from Alméciga-Díaz, C.J. *et al*., 2019 [1].

					**Global response of MPS IVA fibroblasts after CRISPR/nCas9 plus Donor AAVS1:GALNS donor treatment, with respect to untreated cells**				
**Cell ID**	**Mutations***	**Gender**	**Age**	**Trans.**	**GALNS Act. (%)**	**GAGs (FC)**	**LM (FC)**	**mtROS (FC)**	***β*-Hexo (FC)**
**GM00593**	p.R386C p.F285del	F	7	LP	30.5	0.33	0.78	0.63	1.58
				ML	62.1	0.51	0.87	0.79	2.35
**GM01361**	p.R61W p.W405_T406del	M	43	LP	29.7	0.25	0.89	0.84	1.29
				ML	21.1	0.29	0.83	0.78	1.53
**GM00958**	p.A393S	M	12	LP	13.6	0.82	**0.99**	0.93	1.49
				ML	5.1	0.88	**1.04**	**1.10**	**1.31**
**GM01259**	p.A393S p.R94C	F	14	LP	42.1	0.62	0.79	0.77	1.79
				ML	23.3	0.85	0.95	0.62	1.25

Supplementary Figure 1. CRISPR/nCas9 and Donor AAVS1:GALNS vectors. CRISPR/nCas9 corresponds to AIOmCherry Addgene 74120, which contains a mutated version of Cas9 (D10A). Two sgRNA against AAVS1 were cloned into sgRNA 1 and 2. CHI: Cytomegalovirus Promoter plus Hybrid Intron. This vector also encodes for mCherry upstream of Cas9. For Donor AAVS1:GALNS, the vector was constructed as an expression cassette containing the human version of GALNS according to Leal & Alméciga, 2022 [2]. Homologous recombination arms (HRA) for AAVS1 were included for flanking the cassette. **CMV:** Cytomegalovirus promoter. **K:** Kozak element. **P2A:** 2A self-cleaving peptide. **BGC-PA:** Bovine growth hormone polyadenylation signal.

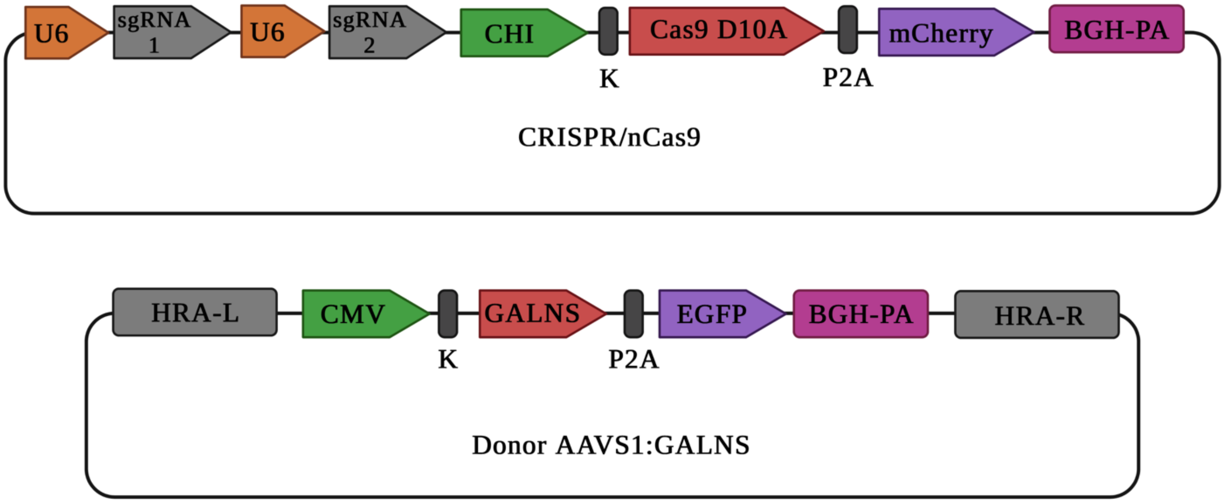

Supplementary Figure 2. Unprocessed *DpnI* digestion gel. Agarose gel shows Ladder (1), undigested DNA (2), naked DNA (3), MNP (4)- and MLPs (5)-coupled plasmid DNA.

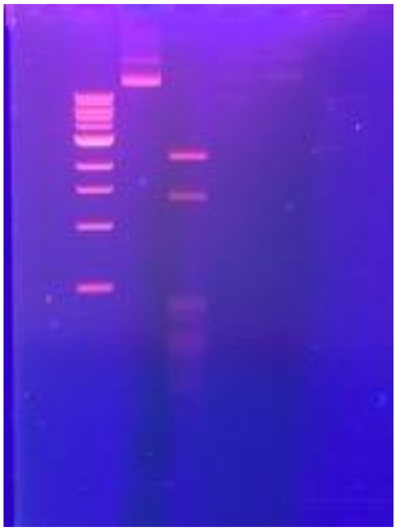

Supplementary Figure 3. Cytotoxicity assays for MPS IVA fibroblasts incubated for 48 hours with 25μg/mL/0.05mg/mL MNPs@Ag-pD/BUF-II:liposome ratio. Tetrazolium reduction assay (MTT, Sigma) and lactate dehydrogenase release (LDH, Roche) were used to determine the percentage of viable cells. In both assays, more than 80% of cell viability was observed for all the different cells.

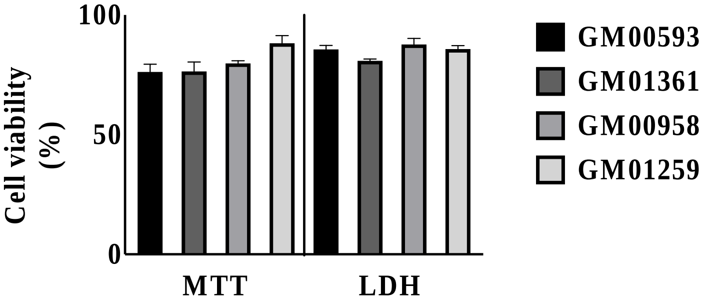

Supplementary Figure 4. Extracellular release of functional GALNS enzyme on GM00593 fibroblasts after CRISPR/nCas9-based genome editing. Note the physiological levels reached for LP. Fibroblasts were transfected either with the Donor AAVS1:GALNS plasmid (Donor) or the CRISPR/nCas9-Donor AAVS1:GALNS plasmids (CRISPR/nCas9:Donor). **p* = *<0.05*. Two-tailed Student's t-test.

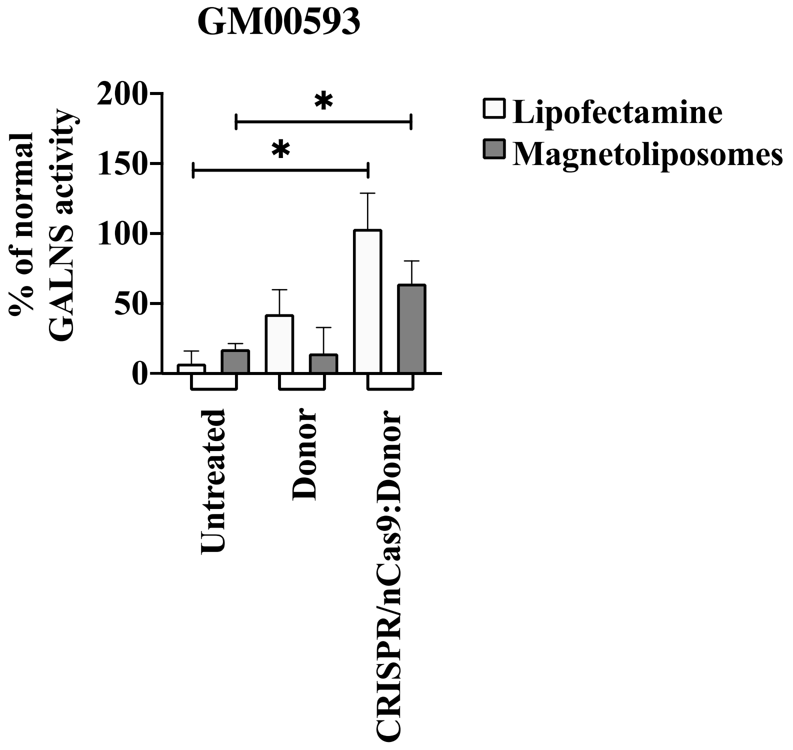

Supplementary Fig. 5. Nitrites determination assay on MPS IVA fibroblasts after CRISPR/nCas9 treatment using LP, and MLPs as carriers. Positive control corresponds to wild-type fibroblasts treated with LPS. Fibroblasts were transfected with the CRISPR/nCas9-Donor AAVS1:GALNS plasmids (CRISPR/nCas9:Donor). *p = <*0.05*. Two-tailed Student’s t-test.

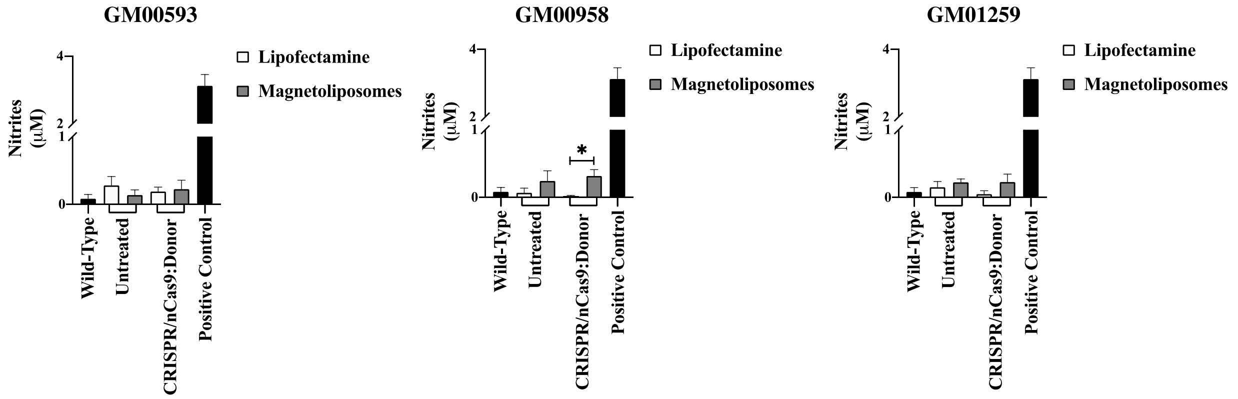

Supplementary References

1. Almeciga-Diaz, C.J., et al., *Identification of ezetimibe and pranlukast as pharmacological chaperones for the treatment of the rare disease mucopolysaccharidosis type IVA.* J Med Chem, 2019. **62**(13): p. 6175-6189.
2. Leal, A.F. and C.J. Alméciga-Díaz, *Efficient CRISPR/Cas9 nickase-mediated genome editing in an in vitro model of mucopolysaccharidosis IVA.* Gene Ther, 2022.
